# Supplementary material for: A Solvent-Mediated Excited-State Intermolecular Proton Transfer Fluorescent Probe for Fe3+ Sensing and Cell Imaging
Source: Molecules. 2022 Jan 14;27(2):516. doi: 10.3390/molecules27020516 (PMC8778147; doi:10.3390/molecules27020516)
Supplement: Supplementary file 1 [file molecules-27-00516-s001.zip › molecules-1551289-supplementary.pdf]

Supplementary data for

A solvent-mediated excited-state intermolecular proton transfer fluorescent probe for Fe^{3+} sensing and cell imaging

You Qian[a], Fuchun Gong[a],* Jiguang Li[b]*, Pan Ma, Hanming Zhu[a], Lingzhi He[a], Jiaoyun Xia[a]

[a]*College of Chemistry and Chemical Engineering, Changsha University of Science and Technology, Changsha, 410114, P. R. China*

[b]*Crop Research Institute, Hunan Academy of Agricultural Sciences, Changsha, 410125, P. R. China*

1. ^{1}H NMR , ^{13}C NMR and FTIR spectrum of 2-APC

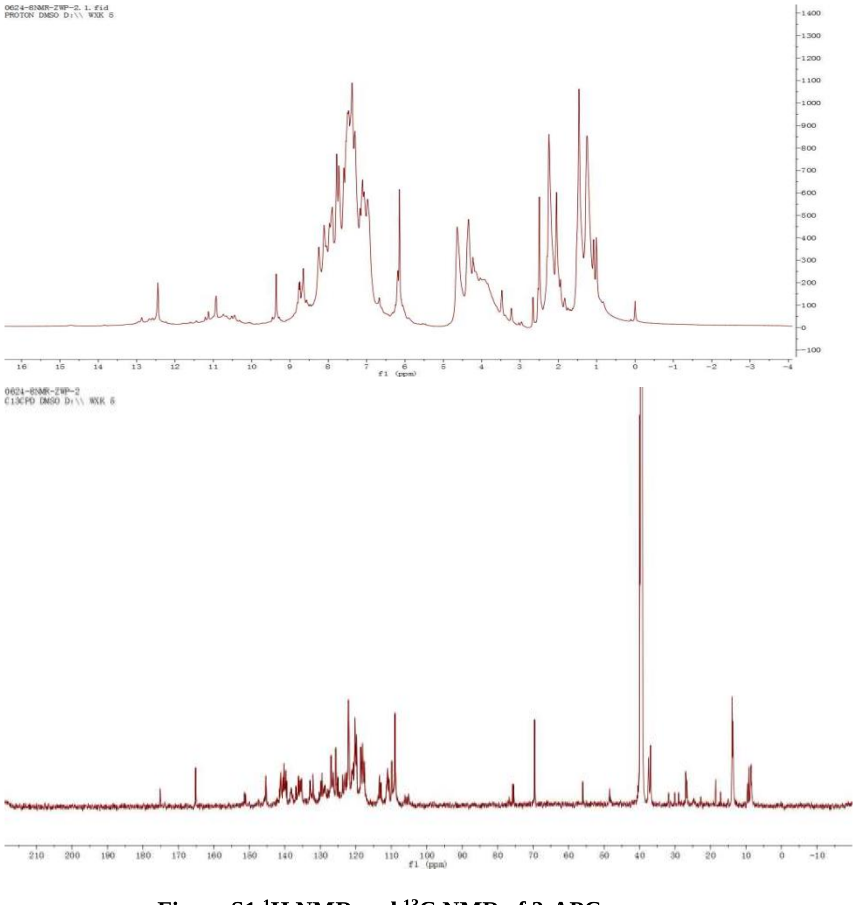

Figure S1 ^{1}H NMR and ^{13}C NMR of 2-APC.

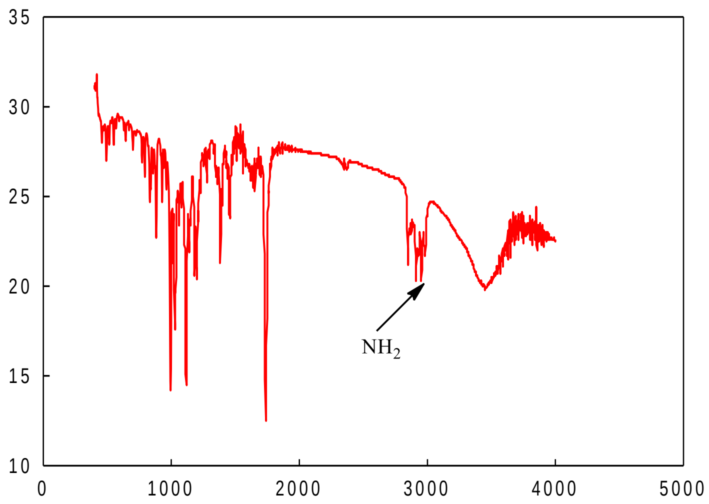

Figure S2 FTIR spectrum for the compound 2-APC.

2. Determination of fluorescence quantum yield

The fluorescence quantum yield (FQY) measurements were carried out using quinine sulfate (FQY=0.54 at 360 nm) in sulfuric acid (0.10 mol.L^{-1}, η =1.33) as the standard substance. The absolute FQY values were estimated corresponding to the following equation:

$$\Phi_u=\Phi_s(I_u/I_s)(A_s/A_u)(\eta^2{}_u/\eta^2{}_s)$$

Φ is fluorescence quantum yield; I is the determined integrated fluorescence intensity; A is the optical density measured at the selected excitation wavelength; η is the refractive index. The subscript "*s*" refers to the standard FQY of the reference quinine sulfate. The subscript "*u*" refers to the unknown FQY of the fluorescent PNPs. In order to minimize re-absorption effect, absorbance in the 1.0 cm fluorescence cuvette was kept under 0.1 at the excitation wavelength of 360 nm.
